# Clinical, genetic and microbiological characterization of pediatric patients with cystic fibrosis in a public Hospital in Ecuador

**DOI:** 10.1186/s12887-020-2013-6

**Published:** 2020-03-06

**Authors:** Yazmina Lascano-Vaca, Esteban Ortiz-Prado, Lenin Gomez-Barreno, Katherine Simbaña-Rivera, Eduardo Vasconez, Alexander Lister, María Emilia Arteaga-Espinosa, Geovanny F. Perez

**Affiliations:** 1 Pediatric Pneumology Service, Pediatric Unit of the Carlos Andrade Marin Specialties Hospital, Quito, Ecuador; 2 One Health Research Group, Universidad de Las Americas, José Queri and Av. de los Granados, Quito, Ecuador; 3 Faculty of Medicine, University of Southampton, Southampton, England; 4 Genetics Department, Gynemedic, Mexico City, Mexico; 5 Division of Pulmonary and Sleep Medicine, Children’s National Health System, Washington, DC USA

**Keywords:** Cystic fibrosis, Epidemiological analysis, Clinical characterization

## Abstract

**Background:**

To carry out a complete clinical, pathological, genetic and microbiological characterization of pediatric patients with molecular confirmed cystic fibrosis (CF) attending the Carlos Andrade Marín Hospital (HCAM) within the study period.

**Methods:**

A cross-sectional analysis of the pediatric population with a confirmed diagnosis of CF disease who attended HCAM, one of the largest tertiary-level hospitals in Ecuador, between 2017 and 2018 was performed. All demographic, clinical and genetic variables were obtained from the electronic medical records (EMR) stored by the hospital.

**Results:**

Forty seven patients with CF were included in the study. Gender distribution was similar between male (48.9%, *n* = 23) and female patients (51.1%, *n* = 24). The Tiffeneau-Pinelli index (FEV_1_/FVC) changed significantly after nine months post-diagnosis (85.55 ± 13.26; *p* < 0.05). The most common pathogenic genetic variants were F508del, found in 52.78% of the cohort (*n* = 19); H609R, found in 36.11% (*n* = 13); g.204099A > C, found in 14.1% (*n* = 7), followed by G85E and the N1303K with 11.11% (*n* = 3) each.

**Conclusions:**

To our best knowledge, this is the first study exploring the clinical, genetic and bacteriological profile of CF’s patients in Ecuador. Within the cohort of patients, an important and unique genetic feature was characterized by the presence of the g.204099A > C and the c.206359C > A homozygous polymorphism as well as the presence of the H609R variant, a mutation only reported among Ecuadorians.

## Background

Cystic fibrosis (CF) is an autosomal recessive disorder caused by a variant in a gene located on the long arm of chromosome 7. The affected gene is the cystic fibrosis transmembrane regulator (*CFTR*), which when mutated produces an abnormal production of the cystic fibrosis transmembrane regulator protein [1–3]. This condition affects many organ systems within the body [4–7], with a clinical presentation potentially including progressive obstructive lung disease, bronchitis, bronchiectasis, mild to severe pancreatic insufficiency, malnutrition and recurrent sinusitis, with infertility also being common in men [7, 8].

CF has become an important public health problem. It has a great impact in terms of healthcare-related costs and on the years of potential life lost (PYLL), resulting in an economic burden for health systems globally [9–12]. CF is one of the most common and lethal genetic diseases, affecting an estimated 70,000 patients from all ethnic groups worldwide [8]. The incidence ranges from 1:2000 to 1:35,000 in newborns, representing more than 1000 new cases of CF that are diagnosed each year before the age of two [7, 8, 12, 13]. Universal newborn screening and early treatment have markedly improved median survival rates in high-income countries (HIC) in comparison to low- and middle-income countries (LMIC) [12, 14–16]. In countries like the United States, Canada and some European countries, average survival curves have improved in the last 30 years, with some patients reaching life expectancies of 50 years or more [17–20]. On the other hand, patients from LMICs often have poorer health outcomes, living on average 10 to 15 years less than individuals with CF from HICs [15, 20, 21]. Zolin et al. revealed that mortality among younger patients (under 18 years of age) reaches 1.3% in LMIC and less than 0.6% in high-middle income countries (HMICs) [22]. In Latin-America, most of the children presenting with related symptoms are misdiagnosed or not diagnosed at all, usually leading to early deaths before reaching the first five years of age [16]. In Latin America, CF’s incidence ranges from 1:6000 to 1:12,000 per every live birth. For instance, Mexico has an incidence of 1:9000, Uruguay at 1:9600 and in Argentina an estimation of 1:6573 per every live birth [23].

In Ecuador, there has been few academic studies on CF. An analysis from Valle et al. [24] determined for the first time, the incidence of the disease in the country at 1:11,252 live births [24]. Although there are a few reports of CF in Ecuador, there are no detailed reports including diagnostic, microbiologic and genetic analysis, as well as pulmonary function data, as recommended by international guidelines such as the North American Cystic Fibrosis Foundation [25–27].

The objective of this study was to conduct a complete clinical, pathological, genetic and microbiological characterization of pediatric patients with molecular confirmed CF attending the Carlos Andrade Marín Hospital within the study period.

## Methods

### Study design

A cross-sectional study of pediatric patients with confirmed cystic fibrosis from the tertiary-level hospital, Carlos Andrade Marín (HCAM), was performed. The data included information from all available aspects of the patients’ charts recorded between June 2017 to July 2018.

### Setting

This study was conducted in the largest capacity hospital of the Social Security Institute (IESS). The HCAM has a clinic system with a multidisciplinary team specialized in management of CF, who receive referrals and admissions from the nearby provinces and cities. The hospital is located in Quito, the capital of Ecuador. During the study period, the clinic received 81 patients, pediatric and adult, with diagnosis of cystic fibrosis.

### Participants

A total of 48 individuals aged 16 or younger with a confirmed diagnosis of cystic fibrosis (positive sweat test accompanied a pathological genetic variant report) were approached for inclusion in this study. One child whose parents did not consent to participate was excluded from the study, leading to 47 pediatric patients being included for analysis. It is important to emphasize that there were two pairs of siblings within the study.

### Treatment

Throughout the study, all patients had the same standard treatment protocol that included: respiratory therapy with inhaled Dornase Alfa and hypertonic saline twice daily, respiratory physiotherapy with prolonged slow expiratory and forced expiratory techniques, and digestive enzymes according to weight to all patients with stool elastase less than 200. For patients with *Pseudomonas* bacteria colonization, tobramycin through inhalation was administered twice daily for 28 days. All additional interventions due to exacerbations were described in the results section.

### Ethical considerations

Informed consent was obtained from every patient and their parents or their legal representatives after disclosing the full purpose of the study. All data were anonymized before further analysis through assigning a study reference number between one and forty-seven, allocated upon patient presentation. The local Institutional Review Board at HCAM approved this study in 2017.

### Data

Demographic, clinical and analytic variables were obtained by reviewing electronic medical records (EMR) from all the patients included in the study in a 12-month period. Demographic information was obtained for analysis, which included the patient’s age, gender, ethnicity, place of residence and educational attainment level. Clinical parameters included family and personal medical history, respiratory/gastrointestinal symptomatology and Shwachman - Kulczycki (SK) disease severity score. The SK scores were considered in the categories of ‘excellent’ (86–100), ‘good’ (71–85), ‘mild’ (56–70), ‘moderate’ (41–55), or ‘severe’ disease severity (≤40). Follow-up was conducted through ongoing scheduled clinician appointments at the hospital. Analytical tests were performed to evaluate pulmonary function using a Spirometer (CareFusion Germany 234 GmbH software) following the ATS/ERS standard criteria. Forced Vital Capacity (FVC), Forced Expiratory Volume in One Second (FEV_1_) and FEV_1_/FVC measurements were used for statistical analysis. In addition, microbiological cultures and sensitivity tests were conducted in every patient. Those children under five years of age had deep throat cultures taken, and for children over five years of age, sputum samples were used to evaluate microbiological colonization. Finally, all patients were analyzed for *CFTR* gene variants by polymerase chain reaction (PCR) and Sanger sequencing by cycling temperature capillary electrophoresis of a panel of 10 specific *CFTR* common variants for the Ecuadorian population. Also, 13 patients had *CFTR* full-length sequencing analysis, which was processed in the United States. Since the full-length sequencing tests were not available in Ecuador and the exportation of the samples were not covered by the public insurance, some patients were not able to obtain the resources to proceed with the full sequence.

### Statistical analysis

Data were analyzed using the software SAS (Version 9.3; SAS Institute Inc., Cary, NC, USA). Patients were categorized into four subgroups: < 5 years, 5 to 9 years, 10 to 14 years old and ≥ 15 years old. Descriptive statistics, such as simple frequencies and means, were used to calculate demographic distribution, clinical findings, cultures and sensitivity of germs, and genetic profiles. For pulmonary function, statistics calculated were a parametric test with Shapiro-Wilk formula and a paired t-test for related samples to evaluate the deterioration of pulmonary function. Statistical significance was determined at p ≤ 0.05.

## Results

### Demographics

Out of 48 pediatric patients that fulfilled the inclusion criteria, 47 were included since one patient withdraw the consent. The sex distribution corresponded to 24 females and 23 males. Age distribution at study period demonstrated that 27.7% (*n* = 13) were under five years, 23.4% (*n* = 11) were between five and nine years, 31.9% (*n* = 15) were between 10 and 14 years and 17.0% (*n* = 8) of patients were 15 years or older, with a median age of 9.2 years (SE +/− 4.98 years). The maximum age was 16 years. In the case of age of diagnosis referred by parents, it was found an age of 5.3 years (SE +/− 5.26 years). Overall, 89.4% (*n* = 42), 6.4% (*n* = 3) and 4.3% (*n* = 2) of patients were auto-identified as mixed race, white and indigenous, respectively.

### Clinical variables

A total of 41 (87.2%) children were referred to the hospital for diagnosis due to persistent respiratory symptoms (cough, recurrent pneumonia, dyspnea on exertion and chest pain). Six patients (12.8%) were referred with persistent gastrointestinal symptoms (including abdominal distention, increased frequency of stools, flatulence or steatorrhea). These symptoms were present at the time of diagnosis, and it was because of these symptoms the individuals had been referred to the hospital for diagnosis. Four patients (8.5%) had positive family history of the disease (Table 1).

**Table 1:** Clinical findings reported among patients with CF in Ecuador

Clinical findings reported at CF Diagnosis				
	< 5 (%)	6 to 10 (%)	11 to 15 (%)	≥ 16 (%)
**Number of individuals (n)**	13	11	15	8
Familiar history with asymptomatic patient	15.4	9.1	6.7	0.0
**Symptoms**				
Persistent respiratory symptoms	69.2	90.9	93.3	100.0
Persistent gastrointestinal symptoms	38.5	0.0	6.7	0.0
**Signs**				
Digital clubbing	0.0	27.3	13.3	25.0
Abnormal liver function test	7.7	0.0	0.0	0.0
Sinus disease	0.0	9.1	6.7	25.0
Malnutrition	23.1	18.2	33.3	25.0
Body mass index percentile (average)	^a^44.8	^a^37.6	^a^37.8	^a^28.3
**Score Shwachman – Kulczycki**				
Excellent	53.9	63.6	33.3	25.0
Good	46.1	27.3	20.0	12.5
Mild	0.0	9.1	46.7	50.0
Moderate	0.0	0.0	0.0	12.5
Severe	0.0	0.0	0.0	0.0
**Comorbidities**				
Cyrstic fibrosis related Diabetes	0.0	0.0	6.7	37.5
Asthma	0.0	36.4	40.0	25.0
Pulmonary Hypertension	0.0	0.0	6.7	12.5
Celiac disease	0.0	0.0	6.7	0.0
Cholelithiasis	0.0	0.0	6.7	0.0
Allergic bronchopulmonary aspergillosis	0.0	9.1	13.3	0.0
Pancreatitis	0.0	18.2	0.0	0.0
Meconium ileus/other intestinal obstruction	0.0	9.1	6.7	0.0

^a^Values expressed in average

Nutritional evaluation included body mass index (BMI) and percentile tables. Malnutrition was found in 25.5% (*n* = 12) of children with the females showing a median BMI of 17.4 kg/m^2^ (percentile 42nd; z-score − 0.42) and males showing an average BMI of 15.8 kg/m^2^ (percentile 28th; z-score − 0.87). Patients not recorded as malnourished had a median BMI that was around the 38th percentile (z-score − 0.62). The Shwachman – Kulczycki score analysis at diagnosis demonstrated that almost half of the patients (44.7%, *n* = 21) had scores of ‘excellent’, followed by ‘good’, ‘mild’ and ‘moderate’ scores in 27.66% (*n* = 13), 25.5% (*n* = 12) and 2.1% (*n* = 1) respectively. Furthermore, no “severe scores” were reported. Asthma was present in 25.5% (*n* = 12) of cases, followed by CF-related diabetes in 8.5% (*n* = 4), meanwhile allergic bronchopulmonary aspergillosis was reported in 6.4% (*n* = 3).

Throughout the study period, a total of 14.9% (*n* = 7) patients had exacerbations that required hospital management with clinicians, with an average of 2.5 hospitalizations per patient and an average inpatient stay of 18 days. Finally, during this study period, there were no deaths reported among the patients.

### Pulmonary function

**Fig. 1:**
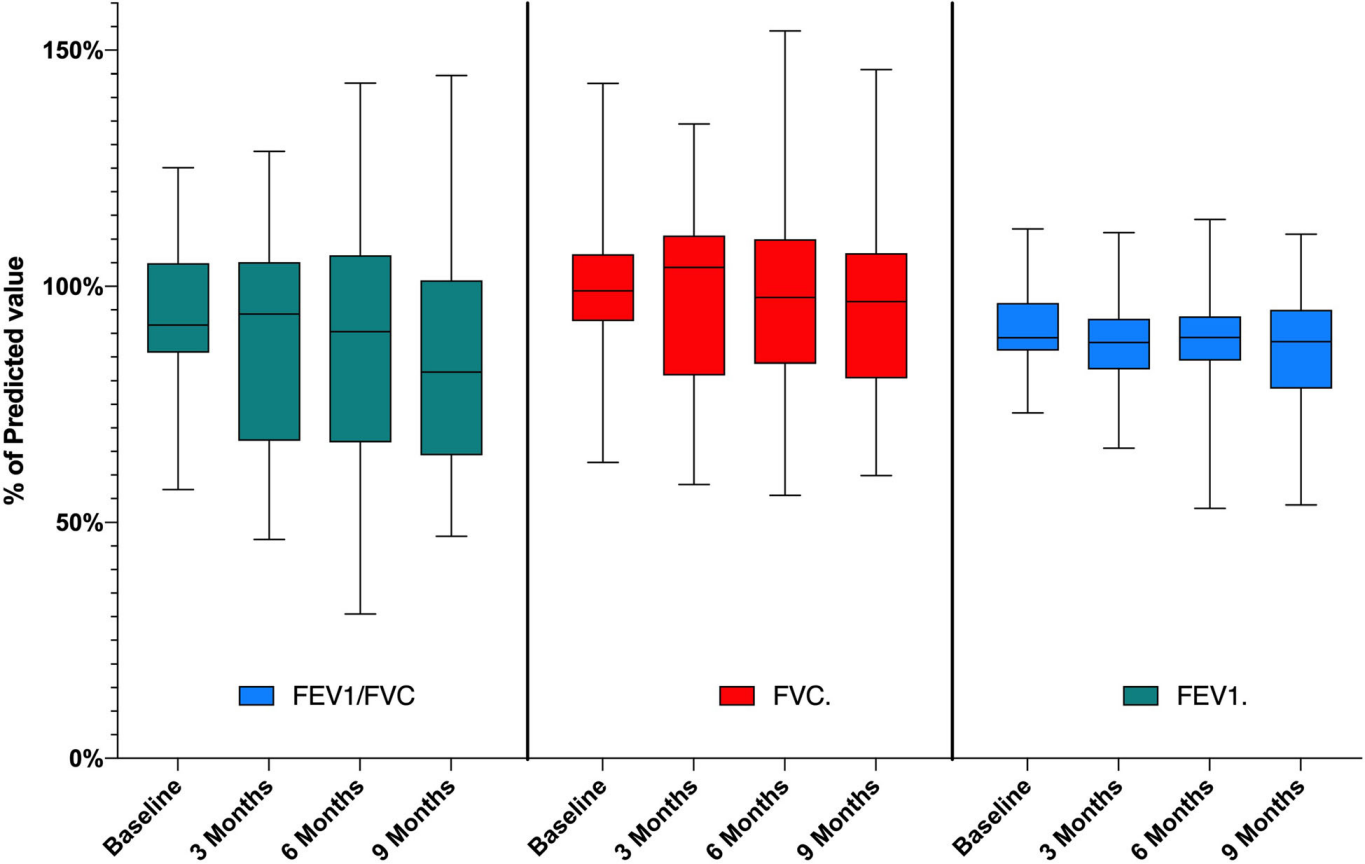
Spirometry results from baseline up to 9 months follow-up in patients from Ecuador. * FEV1/FVC is measured in percentage, while FVC and FEV1 are expressed in percentage of the predicted value

Pulmonary function tests were indicated in 28 patients (59.6%). At diagnosis, spirometry showed values of FEV_1_ with a mean of 92.67% pred. (SD = ± 16.95), FVC with a mean of 100.81% pred. (SD = ± 15.37) and FEV_1_/FVC with a mean of 89.81 (SD = ± 7.69). When comparing the difference between FEV_1_ pulmonary function at diagnosis (baseline) and at follow-up, values show at three months follow-up a non-significant drop in FEV_1_ (Mean = 88.87 ± 22.33; *p* > 0.05) as well as at six months follow-up (Mean = 89.04 ± 27.66; *p* > 0.05) and a significant drop at nine months (Mean = 86.40 ± 24.80; *p* < 0.05). In addition, follow-up mean difference comparison of FVC at three months follow-up was a non-significant drop (Mean = 98.65 ± 18.75; *p* > 0.05) and at six months (Mean = 97.04 ± 22.91; *p* > 0.05), with a significant drop in FVC at nine months (Mean = 95.77 ± 18.94; *p* < 0.05) (Fig. 1).

The differences in pulmonary function by age group and follow-up visit are displayed in Table 2.

**Table 2:** Pulmonary function among Ecuadorian patients with CF divided in three age groups

Age group	Follow-up	*FEV1%pred. (SD)	*FVC %pred. (SD)	FEV1/FVC (SD)
5 to 9 years (*n* = 11)	At diagnosis	105.3 (13.1)	106,3 (15.9)	98,5 (8.2)
	3-month	95,8 (27.2)	100,8 (22.3)	92,5 (10.9)
	6-month	104,7 (30.7)	109,4 (25.3)	93,7 (11.9)
	9-month	105,3 (29.5)	107,0 (23.5)	95,3 (10.9)
10 to 14 years (*n* = 15)	At diagnosis	87,7 (12.8)	97,5 (11.5)	87,9 (4.3)
	3-month	87,1 (20.9)	97,0 (18.5)	88,0 (6.6)
	6-month	86,6 (22.7)	94,1 (20.1)	88,0 (8.3)
	9-month	80,4 (18.3)	92,3 (16.3)	81,7 (11.9)
≥ 15 years (*n* = 8)	At diagnosis	90,4 (19.9)	99,6 (18.3)	87,6 (8.0)
	3-month	85,8 (22.3)	100,0 (18.2)	83,0 (11.6)
	6-month	76,9 (31.9)	90,1 (25.3)	72,8 (29.4)
	9-month	79,3 (25.8)	91,3 (17.2)	83,8 (15.1)

*FEV1 (Forced expiratory ventilation at first second) and FVC (Forced vital capacity) are expressed as a percentage of the predicted values (% pred.), while FEV1/FVC is expressed in percentage

The comparison among different times relative to the mean differences in FEV_1_/FVC at three (Mean = 87.86 ± 9.36; *p* > 0.05), six (Mean = 87.89 ± 11.63; *p* > 0.05) and nine months (Mean = 85.55 ± 13.26; *p* < 0.05) is displayed in Table 2.

### Microbiology

**Fig. 2:**
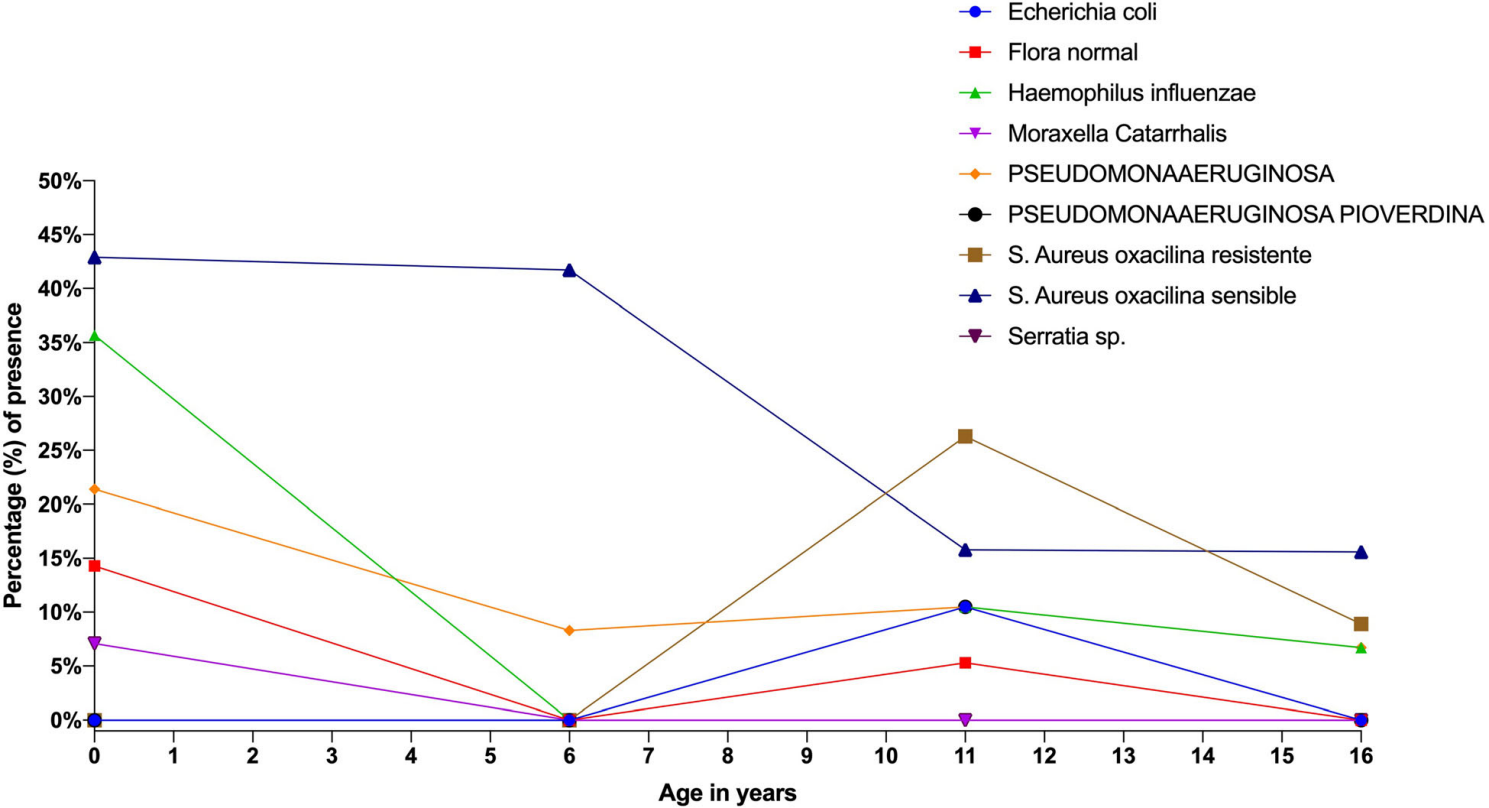
Presence of different microorganism per age group according to the bacteriological culture results

Bacteriological cultures and antibiograms were obtained every three months after the date of diagnosis. The most common isolated organism was *Staphylococcus aureus* (oxacillin sensitive) in 25.9% of the samples, followed by *Haemophilus influenzae* in 17.7% and *Pseudomonas aeruginosa* in 12.5%. Age group analysis showed *Haemophilus influenza* as the most common pathogen in patients younger than 10 years, while *Moraxella Catarrhalis* decreased in frequency in patients older than 10 years. *Staphylococcus aureus* (oxacillin sensitive and resistant) and *Pseudomonas aeruginosa* became more common in patients older than 10 years (Fig. 2)*.*

A total of 120 susceptibility tests were performed; four species of gram-positive bacteria (*S. aureus, Coagulase-Negative Staphylococcus, Streptococcus pneumoniae* and *M. catarrhalis*) were the most common, with *S. aureus* showing the largest frequency (*n* = 95) among cultures, being most of them highly susceptible to sulfas, vancomycin, linezolid and ciprofloxacin while erythromycin was not effective (resistant) in most cases. Other cultures reported several types of gram-negative organisms, while only 10% of the isolate’s cultures reported gram-positive organisms (Table 3). The less common pathogens found among cultures were *Acinetobacter spp, Achromobacter spp, Serratia spp and Raultella planticola.* These microorganisms were found only once; therefore, susceptibility tests were not performed. The most frequent gram-negative pathogen was *Pseudomonas spp,* found in 29.2% of cultures. *Pseudomonas spp* were susceptible to Cefepime, Ceftazidime, Meropenem, Imipenem and Piperacillin/Tazobactam, but showed resistance in almost 20% of cases against Amikacin and Gentamicin, as well as Ciprofloxacin in less than 10% of isolated cultures. *Haemophilus spp* was found in 16.7% of tests, with the bacteria demonstrating high sensitivity to Ampicillin/Sulbactam, Azithromycin, Ceftriaxone and Cefuroxime, however, was resistant to Co-trimoxazole and Ampicillin in less than 50 and 15% of tests respectively. *Enterobacter spp* was isolated in 10.8% of the samples and showed to be highly sensitive to Ceftazidime and Gentamicin as well as to Ceftriaxone and Piperacillin/Tazobactam (Table 3).

**Table 3:** Bacteriological culture results from Ecuadorian patients with CF

	Microorganisms	Isolations (n)	Antibiotics	Susceptibility (n)	Susceptibility (%)	Resistance (n)	Resistance (%)	Non-described (n)	Non-described (%)
Gram-positive	*Staphylococcus aureus*	95	Ciprofloxacin	55	57.89	4	4.21	36	37.89
			Clindamycin	46	48.42	38	40.00	11	11.58
			Erythromycin	19	20.00	61	64.21	15	15.79
			Gentamicin	64	67.37	15	15.79	16	16.84
			Linezolid	55	57.89	0	0.00	40	42.11
			Oxacillin	40	42.11	39	41.05	16	16.84
			Co-trimoxazole	74	77.89	1	1.05	20	21.05
			Vancomycin	51	53.68	0	0.00	44	46.32
	Coagulase-Negative Staphylococcus	3	Clindamycin	0	0.00	2	66.67	1	33.33
			Gentamicin	2	66.67	0	0.00	1	33.33
			Erythromycin	0	0.00	2	66.67	1	33.33
			Oxacillin	1	33.33	1	33.33	1	33.33
			Penicillin	0	0.00	2	66.67	1	33.33
			Co-trimoxazole	2	66.67	0	0.00	1	33.33
			Vancomycin	2	66.67	0	0.00	1	33.33
	*Streptococcus pneumoniae*	7	Ceftriaxone	4	57.14	0	0.00	3	42.86
			Clindamycin	2	28.57	1	14.29	4	57.14
			Penicillin	4	57.14	2	28.57	1	14.29
			Co-trimoxazole	2	28.57	3	42.86	2	28.57
	Corynebacterium spp	2	Doxycycline	1	50.00	0	0.00	1	50.00
			Levofloxacin	1	50.00	0	0.00	1	50.00
	*Escherichia coli*	11	Amikacin	6	54.55	3	27.27	2	18.18
Gram negatives			Ampicillin/sulbactam	0	0.00	8	72.73	3	27.27
			Cefepime	1	9.09	9	81.82	1	9.09
			Ceftazidime	1	9.09	9	81.82	1	9.09
			Ciprofloxacin	1	9.09	7	63.64	3	27.27
			Gentamicin	4	36.36	4	36.36	3	27.27
			Imipenem	10	90.91	0	0.00	1	9.09
			Piperacillin/tazobactam	4	36.36	3	27.27	4	36.36
	Enterobacter spp	13	Ceftazidime	10	76.92	0	0.00	3	23.08
			Ceftriaxone	8	61.54	0	0.00	5	38.46
			Gentamicin	10	76.92	0	0.00	3	23.08
			Piperacillin/tazobactam	8	61.54	0	0.00	5	38.46
	Haemophilus spp	20	Ampicillin	13	65.00	3	15.00	4	20.00
			Ampicillin/sulbactam	14	70.00	1	5.00	5	25.00
			Azithromycin	15	75.00	0	0.00	5	25.00
			Ceftriaxone	13	65.00	0	0.00	7	35.00
			Cefuroxime	13	65.00	1	5.00	6	30.00
			Co-trimoxazole	6	30.00	9	45.00	5	25.00
	Klebsiella spp	5	Amikacin	3	60.00	0	0.00	2	40.00
			Ampicillin/sulbactam	3	60.00	0	0.00	2	40.00
			Cefepime	3	60.00	0	0.00	2	40.00
			Ceftazidime	4	80.00	0	0.00	1	20.00
			Ciprofloxacin	3	60.00	0	0.00	2	40.00
			Imipenem	3	60.00	0	0.00	2	40.00
	Moraxella spp	7	Amoxicillin/clavulanate	4	57.14	0	0.00	3	42.86
			Ampicillin	0	0.00	5	71.43	2	28.57
			Ampicillin/sulbactam	5	71.43	1	14.29	1	14.29
			Azithromycin	6	85.71	0	0.00	1	14.29
			Cefuroxime	6	85.71	0	0.00	1	14.29
	Pseudomonas spp	35	Amikacin	11	31.43	6	17.14	18	51.43
			Cefepime	27	77.14	3	8.57	5	14.29
			Ceftazidime	31	88.57	1	2.86	3	8.57
			Ciprofloxacin	21	60.00	4	11.43	10	28.57
			Gentamicin	16	45.71	8	22.86	11	31.43
			Imipenem	20	57.14	1	2.86	14	40.00
			Meropenem	26	74.29	1	2.86	8	22.86
			Piperacillin/tazobactam	25	71.43	2	5.71	8	22.86

### Genetic analysis

The genetic tests were comprehensively compared and correlated with the information available from the ClinVar and the Single Nucleotide Polymorphism (dbSNP) databases (Table 4). Among children attending HCAM, 36 out of 47 patients underwent *CFTR* sequence analysis. A total of 23 patients had a specifically targeted variant panel, and 13 of them had full-length gene sequencing.

**Table 4:** Classification of the genetic variants found among Ecuadorian children with CF

Genetic identification	Nucleotide identification	Protein identification (change)	dbSNP ID	Clinical Significance	Molecular consequence	Class of allele mutation	Clinical classification	CFTR 2 database patient reports
**g.19395G > A**	**–**	**–**	Not reported	Not reported	Not reported	Non classified	Non classified	Not reported
**g.43555G > C**	**–**	**–**	Not reported	Not reported	Not reported	Non classified	Non classified	Not reported
**g.43575G > C**	**–**	**–**	Not reported	Not reported	Not reported	Non classified	Non classified	Not reported
**g.43580G > T**	**–**	**–**	Not reported	Not reported	Not reported	Non classified	Non classified	Not reported
**g.43583A > G**	**–**	**–**	Not reported	Not reported	Not reported	Non classified	Non classified	Not reported
**g.43592 T > C**	**c.164 + 12 T > C**	**–**	rs121908790	Uncertain	Intron variant	Non classified	Non classified	Not reported
**g.43594A > G**	**–**	**–**	Not reported	Not reported	Not reported	Non classified	Non classified	Not reported
**g.48340G > A**	**c.254G > A**	**p.Gly85Glu (G85E)**	rs75961395	Pathogenic	Missense variant	Class II	A group	584
**g.73512G > A**	**c.509G > A**	**p.Arg170His (R170H)**	rs1800079	Pathogenic	Missense variant	Non classified	Non classified	11
**g.70332G > T**	**c.489 + 1G > T**	**621 + 1G > T**	rs78756941	Pathogenic	Splice donor variant	Class I	A group	1293
**g.74534G > C**	**c.1624G > T**	**p.Gly542Ter (G542X)**	rs113993959	Pathogenic	Nonsense variant	Class I	A group	3489
**g.206154C > T**	**c.869 + 11C > T**	**–**	rs1800503	Benign	Intron variant	Non classified	Non classified	Not reported
**g.79435G > T**	**c.988G > T**	p.Gly330Ter (G330*)	rs79031340	Pathogenic	Nonsense variant	Non classified	Non classified	23
**g.98696A > G**	**c.1408=**	**p.Val470 = (M470V)**	rs213950	Benign	Missense variant	Non classified	C group	209
**g.98808_98811delTCT**	**c.1521_1523del**	**p.Phe508del (F508del)**	rs113993960	Pathogenic	Inframe variant	Class II	A group	65,046
**g.131210A > G**	**c.1826A > G**	**p.His609Arg (H609R)**	rs397508310	Pathogenic	Missense variant	Non classified	Non classified	9
**g.117592218_117592219dup**	**c.2052dupA**	**p.Gln685fs (Gln685Thrfs)**	rs121908746	Pathogenic	frameshift variant	Non classified	Non classified	324
**g.117592382del**	**c.2215del**	**p.Val739fs (2347delG)**	rs397508353	Pathogenic	Frameshift variant	Non classified	Non classified	38
**g.134218 T > G**	**c.2562 T > G**	**p.Thr854 = (T854T)**	rs1042077	Benign	Synonymous variant	Non classified	Non classified	36
**g.142999 G > A**	**c.2908G > A**	**p.Gly970Ser (G970S)**	rs397508453	Uncertain	Missense variant	Non classified	Non classified	10
**g.143018G > T**	**–**	**–**	Not reported	Not reported	Not reported	Non classified	Non classified	Not reported
**g.149918 T > A**	**–**	**–**	Not reported	Not reported	Not reported	Non classified	Non classified	Not reported
**g.74629 T > C**	**c.3294G > A**	**p.Trp1098Ter (W1098X)**	rs397508533	Pathogenic	Nonsense variant	Non classified	Non classified	9
**g.181807A > G**	**c.3870A > G**	**p.Pro1290 = (P1290P)**	rs1800130	Benign	Synonymous variant	Non classified	Non classified	Not reported
**g.192094C > G**	**c.3909C > G**	**p.Asn1303Lys (N1303K)**	rs80034486	Pathogenic	Missense variant	Class II	A group	2147
**g.204099A > C**	**–**	**–**	Not reported	Not reported	Not reported	Non classified	Non classified	Not reported
**g.129569G > A**	**c.1680-1G > A**	**1812-1G > A**	rs121908794	Pathogenic	Splice acceptor variant	Non classified	Non classified	31
**g.206271 G > A**	**c.4387C > T**	p.Gln1463Ter (Q1463*)	rs886044425	Uncertain	Nonsense variant	Non classified	Non classified	Not reported
**g.206359C > A**	**–**	**–**	Not reported	Not reported	Not reported	Non classified	Non classified	Not reported

The most common pathogenic variant reported was F508del, found in 52.8% (*n* = 19) of tested patients. The variant was found in homozygous state in four patients and in heterozygous state in 15 patients. The second most common variant reported was H609R, found in 13 patients (36.1%), most of them in heterozygous state (*n* = 11), while homozygous state was only found among indigenous patients, and may be related to a founder effect [28]. The third most common pathogenic variants were G85E and N1303K, both found in 11.1% of the patients. Less common variants included the W1098X, G542X and the R170H (Table 5).

**Table 5:** Molecular findings among pediatric patients diagnosed with CF in a tertiary level hospital in Ecuador

Genetic identification	Nucleotide identification	Protein identification (change)	Number of reports	Heterozygosis	Homozygosis
g.19395G > A	**–**	**–**	2	–	2
g.43555G > C	**–**	**–**	1	1	–
g.43575G > C	**–**	**–**	1	1	–
g.43580G > T	**–**	**–**	1	1	–
g.43583A > G	**–**	**–**	1	1	–
g.43592 T > C	c.164 + 12 T > C	–	2	2	–
g.43594A > G	–	–	1	1	–
g.48340G > A	c.254G > A	p.Gly85Glu (G85E)	4	4	–
g.73512G > A	c.509G > A	p.Arg170His (R170H)	1	1	–
g.70332G > T	c.489 + 1G > T	621 + 1G > T	1	1	–
g.74534G > C	c.1624G > T	p.Gly542Ter (G542X)	2	2	–
g.206154C > T	c.869 + 11C > T	–	4	3	1
g.79435G > T	c.988G > T	p.Gly330Ter (G330*)	1	–	1
g.98696A > G	c.1408=	p.Val470 = (M470V)	6	3	3
g.98808_98811delTCT	c.1521_1523del	p.Phe508del (F508del)	19	15	4
g.131210A > G	c.1826A > G	p.His609Arg (H609R)	13	11	2
g.117592218_117592219dup	c.2052dupA	p.Gln685fs (Gln685Thrfs)	1	1	–
g.117592382del	c.2215del	p.Val739fs (2347delG)	1	1	–
g.134218 T > G	c.2562 T > G	p.Thr854 = (T854T)	1	1	–
g.142999 G > A	c.2908G > A	p.Gly970Ser (G970S)	1	1	–
g.143018G > T	–	–	1	1	–
g.149918 T > A	–	–	1	1	–
g.74629 T > C	c.3294G > A	p.Trp1098Ter (W1098X)	3	3	–
g.181807A > G	c.3870A > G	p.Pro1290 = (P1290P)	1	1	–
g.192094C > G	c.3909C > G	p.Asn1303Lys (N1303K)	4	4	–
g.204099A > C	–	–	7	–	7
g.129569G > A	c.1680-1G > A	1812-1G > A	1	1	–
g.206271 G > A	c.4387C > T	p.Gln1463Ter (Q1463*)	2	2	–
g.206359C > A	–	–	4	–	4

We found some variants that had already been reported in previous reports and also some not previously reported, including g.204099A > C in 19.4% (*n* = 7), followed by M470V in 16.7% (*n* = 6), c.869 + 11C > T in 11.1% (*n* = 4) and the g.206359C > A in 11.1% (*n* = 4) of cases. Finally, the less commonly reported variants were the g.19395G > A, c.164 + 12 T > C, Q1463* and other particular variants as shown in Table 5.

### Ecuadorian genetic profile

Seven patients who were compound heterozygous for different variants also had other multiple allelic combinations never seen before, such as the g.204099A > C polymorphism, in the seven of them in homozygous state. This polymorphism g.204099A > C has only been reported in Ecuadorian populations to date, establishing its role as a predisposing genetic factor in positive cases (Table 6).

**Table 6:** Predisposing genetic factors in CF positive cases in a cohort of Ecuadorian children attending the HECAM hospital

Patient ID	Mutation	State	Age group	Z Score (mean)	Sweat test (mean)	Prevalent bacteria	Fev1 (mean)	FVC (mean)	Fev1% (mean)	Hospital Admissions	Inpatient days
All population	Tables 4 and 5		9,2 (mean)	-0,62	96,78	Table 3	90,45	98,78	87,73	2,5 (mean)	18 (mean)
3	H609R	Heterozygous	5 to 10 years	1	120	Staphylococcus aureus oxacillin resistant	105,22	104,42	99,38	–	–
	c.204099A > C	Homozygous									
10	c.19395G > A	Homozygous	10 to 15 years	−3,15	110,5	Staphylococcus aureus oxacillin susceptible	91,52	96,45	93,45	–	–
	M470V	Homozygous									
	H609R	Homozygous									
	c.204099A > C	Homozygous									
14	F508del	Heterozygous	10 to 15 years	−1,18	118	Staphylococcus aureus oxacillin resistant	67,45	73,35	78,13	3	18
	W1098X	Heterozygous									
	c.204099A > C	Homozygous									
	c.206359C > A	Homozygous									
	c.43555G > C	Heterozygous									
	c.43592 T > C	Heterozygous									
	c.869 + 11C > T	Heterozygous									
17	T854T	Homozygous	Less than 5 years	0,26	87	Staphylococcus aureus oxacillin resistant	Patient less than 5 years			–	–
	c.204099A > C	Homozygous									
	R170H	Heterozygous									
	G330E	Homozygous									
	Q1463Q	Heterozygous									
25	c. 43580G > T	Heterozygous	10 to 15 years	0,05	86	Staphylococcus aureus oxacillin resistant	93,15	102,58	89,3	1	21
	c.204099A > C	Homozygous									
	G85E	Heterozygous									
	H609R	Heterozygous									
	M470V	Homozygous									
	c.206359C > A	Homozygous									
	c.43583A > G	Heterozygous									
	c.43594A > G	Homozygous									
	c.74629 T > C	Heterozygous									
41	c.143018G > T	Heterozygous	5 to 10 years	−0,54	83,5		93,9	92,56	100,8	–	–
	c.204099A > C	Homozygous				Moraxella spp					
	c.206359C > A	Homozygous				Haemophilus spp					
	M470V	Homozygous									
	G970S	Heterozygous									
45	c.19395G > A	Homozygous	10 to 15 years	−2,16	67	Staphylococcus aureus oxacillin susceptible	72,28	88,58	82,93	–	–
	c.74534G > C	Heterozygous									
	P1290P	Heterozygous									
	M470V	Heterozygous									
	Q1463Q	Heterozygous									
46	c.204099A > C	Homozygous	Less than 5 years	0,33	84,5	Haemophilus spp	Patient less than 5 years			–	–
	c.206359C > A	Homozygous				Moraxella spp					
	M470V	Heterozygous									

## Discussion

CF is an autosomal recessive disorder seen worldwide, with typically a higher prevalence in Caucasian populations [11]. A significant problem in developing countries is that CF patients have a shorter life expectancy, with many patients diagnosed at a later stage in life once symptoms of the disease have manifested [29]. This study has shown that the average age of diagnosis is after the age of nine, with only 38.3% of patients diagnosed before five years of age. Later stage diagnosis is common among developing countries, while in high-income countries, more than 95% of the patients are diagnosed during the first year of life [11, 30].

In the sample of patients, respiratory symptoms (cough, recurrent pneumonia, dyspnea on exertion and chest pain) were most commonly described, followed by gastrointestinal symptoms including abdominal distention, increased frequency of stools, flatulence and steatorrhea. The clinical presentation in Ecuadorian patients appears to be similar to those reports from other Latin-American countries [23, 31]. As often reported, BMI values are often low, and malnutrition has manifested. In our analysis, we found that the patients had an average BMI of 16.6 kg/m^2^, with girls showing greater average BMI values (17.4 kg/m^2^) compared to the boys (15.8 kg/m^2^). Culhane et al. reported higher BMI values for girls being slightly lower (21 kg/m^2^) than boys (22 kg/m^2^), probably due to increased energy losses, increased energy needs or differences in inadequate calorie intake among boys [32]. Despite the later diagnosis of the disease, malnutrition was presented in only a quarter of our patients. The Shwachman-Kulczycki scores of ‘good’ or ‘excellent’ were seen in more than 70% of patients. This grading system is often used to track progress in these types of patients [33, 34].

In terms of comorbidities, a diagnosis of asthma in CF patients is difficult due to overlapping respiratory symptoms presenting in patients. Around 20–30% of the patients with CF can have a concomitant clinical diagnosis of Asthma in population studies [35, 36]. Our results show that 33% of our patients had this comorbidity, which is compatible with previous works.

The FEV_1_, FVC and the Tiffeneau-Pinelli index were within the normal range in the majority of patients. Mild lower airway obstruction was seen in all age groups, which is an interesting finding since lung function would have been expected to be lower in patients with a delayed CF diagnosis, as reported elsewhere [34]. Lung function during follow-up at nine months post-diagnosis was shown to be preserved, which was likely related to the quality of management, which includes correct antibiotic management and appropriate clinical follow-up schemes [28].

Preventing complications is essential for this type of disease management. Periodical cultures had proven to be an important clinical step to take when looking to prevent further complications and implement early treatment when needed. In our report, the most common microorganism reported was *Staphylococcus aureus,* with varying antibiotic sensitivity profiles. The main differences were displayed in terms of age groups, showing a lower incidence of *Moraxella catharralis* and *Haemophilus influenzae* among cultures from patients over 10 years, while these patients had an increase in the number of positive cultures for *S. aureus* and *Pseudomonas aeruginosa*, an observation previously reported elsewhere [37]. The presence of *P. aeruginosa* within the respiratory tract is often used as a positive marker for severity among children and adults with CF [38, 39]. This type of infection accelerates lung-function deterioration and increases the cost of treatments [40, 41]. In our results, *P. aeruginosa* was detected in 12.5% of airway cultures, and those patients were treated immediately with a double regimen of broad-spectrum intravenous (IV) antibiotics.

An important finding of our report was the presence of the H609R variant (caused by the transition of adenosine to guanine at nucleotide 1958 - exon 13). To our best knowledge, this type of variant has only been reported among Andeans’ offspring, as a likely damaging variant [27, 42–44]. Among the 13 patients (38.23%) with a complete sequence, only two patients displayed a homozygous state, while six had a compound heterozygous profile with the presence of the F508del variant [27, 44].

The other variants had been previously described in Ecuadorian patients with CF [27, 44]. Nefzi et al. analyzed Latin American CF patients and found four common variants: F508del (31.37%), G542X (1.96%), G85E (1.96%) and N1303K (1.96%), with 63.7% of Ecuadorian CF variants remaining unidentified [42]. In the second report, in order of frequency, the variants reported were F508del (37.1%), G85E (8.9%), G542X (2.4%), N1303K (2.4%), with a detection rate of 53.22% of the total of CF patients studied. All four of these variants were found in our tested patients in the following detection percentage: 52.77, 5.56, 11.11, and 11.11%, respectively.

*CFTR* exhibits an important allelic heterogeneity, a situation in which different variants in the same gene produce variations in clinical manifestations, with this heterogeneity known to be related to ethnic origin, as we can exemplify with H609R variant which has only reported in Hispanic offspring [27, 42–44]. The other common variants G542X, G85E and N1303K have been consistently found in Ecuadorian patients, with frequencies greater than that of Caucasian variant panels.

In seven of the patients, the polymorphism c.204099A > C was reported, all in homozygous state, this polymorphism has been reported only in Ecuadorian populations [27], with most of these patients also showing known pathological variants. This combination of polymorphisms and variants show a variety of phenotypes in patients, such as nutritional compound, microbiological cultures, respiratory spirometry values or as a combined effect. As Polizzi et al. described, there is a direct relation between phenotype with the specific variant panel [45]. The most severe cases with c.204099A > C polymorphism were between 10 and 15 years old and related to H609R and F508del/W1098X variants. In one of these patients, a poor nutrition status was seen, and in the second there were complications associated with respiratory deterioration. In addition, Moya et al. found in H609R homozygous Ecuadorian patients a severe clinical presentation of CF [44]. This contrasts with our first patient that had a heterozygous alteration with an important nutritional implication. Sebro et al. described F508del in the heterozygous compound, which showed a statistical association between variant and sweat chloride level, pancreatic function and Pseudomonas infection risk [46], also seen in our study. Finally, CFTR2 databases describe that patients with the W1098X variant have a predictive value in the range 62–100% [47], which was also present in our patient. The previous studies did not include patients that were included in this study, as it was confirmed with the patient and their carers that participation in past studies had not taken place.

In four of our patients, the polymorphism c.206359C > A was reported in homozygous state. All were related to F508del/W1098X, G85E/H609R, and G970S variants, however, the first case was described in combination with c.204099A > C polymorphism as opposed to c.206359C > A. In the second patient, there was no marked nutritional alteration, with sweat testing measures showing a low value, the respiratory function was partially conserved, and only one hospital admission was seen. Decaesteker et al. describe that patients with G85E had a significantly higher sweat chloride, higher prevalence of pancreatic insufficiency, worse current weight for height and higher prevalence of chronic *P. aeruginosa* colonization. However, these findings are not highly concordant with our patient [45]. The last case demonstrated slight malnutrition, common bacteria colonization for the age and normal respiratory function. Therefore, there was phenotypic concordance, but there were no studies available about the clinical involvement of G970S variant in the available literature.

During genetic analysis there was a unique case with variant c.204099A > C and c.206359C > A in combination with benign polymorphism M470V. This patient showed only sweat test positive, but there was no malnutrition, microbiological alteration nor complications during the study period. However, sequential spirometry test results were unavailable due to the participant’s age. M470V has been described as a benign polymorphism with slight respiratory problems and recurrence, which does not show on positive on sweat test [48]. The unique presence of that polymorphism combination (c.204099A > C, c.206359C > A and 470 V), in addition to the unexpected positive sweat test, make this case a unique opportunity for further investigation and follow-up in order to find more information about its phenotype expression, its management and its prognosis.

### Bias

Selection bias was present as only cases who gave informed consent were included. Every eligible pediatric CF patient was approached; however only those patients that agreed alongside parents or guardians who agreed to participate were involved in the study. This was a single location study, so results may not generalizable for the whole Ecuadorian population. Information bias was reduced by using standardized medical records from the hospital, which were reviewed by two researchers to reduce any errors in reporting and were transcribed in two different databases that later were reconciled. Any discrepancies between the two reviewers were escalated to the study lead researcher for a resolving discussion. Diagnostic bias was reduced through only including patients with a confirmation of CF through a positive sweat test as well as genetic confirmation in the study.

### Limitations

This study was not exempt from limitations. Patients were sampled from one hospital in Ecuador; therefore, a complete extrapolation to the entire Ecuadorian population might be inaccurate. To have a better understanding of the clinical, genetic and microbiological features of CF in Ecuador, a larger sample size, over multiple hospitals around the country would be needed. Another limitation was the access to funding and equipment. Due to the lack of resources and finance, only 36 of the 47 patients underwent a genetic analysis, a selection that was allocated on a first-come, first-served basis, a decision made according to the presence of available funding. In addition, the lack of a quality asthma research center made it difficult for doctors to test for bronchial hypersensitivity according to international standards, tests that would have been valuable for this type of research.

## Conclusions

To our best knowledge, this is the first study exploring the clinical, genetic and bacteriological profile of CF’s patients in Ecuador. Due to the lack of universal screening in the country, a large proportion of individuals are being diagnosed or misdiagnosed later than expected, jeopardizing their treatments and prognosis. The bacteriological results demonstrated that *S. aureus* was the most common pathogen found within the cultures, most of them showing susceptibility to sulfas, vancomycin, linezolid and ciprofloxacin.

Lastly, the current cohort of patients showed an important and unique genetic feature characterized by the presence of the g.204099A > C and the c.206359C > A homozygous polymorphism, as well as the presence of the H609R variant, a mutation only reported among Andeans dwellers.

## Data Availability

The datasets used during the current study are available from the corresponding author on reasonable request. His email is e.ortizprado@gmail.com.

## Abbreviations

BMI: Body Mass Index
CF: Cystic Fibrosis
CFTR: cystic fibrosis transmembrane regulator
dbSNP: Single Nucleotide Polymorphism Database
EMR: Electronic Medical Records
FEV_1_: Forced Expiratory Volume in One Second
FVC: Forced Vital Capacity
HCAM: Carlos Andrade Marin Hospital
HIC: High Income Countries
IESS: Ecuadorian Institute of Social Security
IV: Intravenous
LMIC: Low- and Middle-Income Countries
PCR: Polymerase Chain Reaction
REGLAFQ: Registro Latinoamericano de Fibrosis Quistica
SK: Shwachman – Kulczycki
